# Reduced perfusion density of superficial retinal capillary plexus after intravitreal ocriplasmin injection for idiopathic vitreomacular traction

**DOI:** 10.1186/s12886-019-1119-9

**Published:** 2019-05-10

**Authors:** Lorenzo Iuliano, Giovanni Fogliato, Roberta Colombo, Riccardo Sacconi, Giuseppe Querques, Francesco Bandello, Marco Codenotti

**Affiliations:** Department of Ophthalmology, San Raffaele Scientific Institute, Vita-Salute University, Via Olgettina 60, 20132 Milan, Italy

**Keywords:** Ocriplasmin, OCT angiography, Vitreomacular traction, Perfusion density, Foveal avascular zone

## Abstract

**Background:**

To investigate, using optical coherence tomography angiography (OCT-A), changes in perfusion density and in the foveal avascular zone (FAZ) in eyes with idiopathic vitreomacular traction (VMT) after ocriplasmin injection.

**Methods:**

In this pilot study, we enrolled sixteen VMT eyes treated with intravitreal ocriplasmin injection. Sixteen healthy eyes were considered as controls. Macular perfusion density in 3 plexuses [superficial capillary plexus (SCP), deep capillary plexus (DCP), choriocapillaris (CC)] was calculated at baseline and at 1 month after injection.

**Results:**

After injection, VMT anatomically resolved in 9 eyes (56.2%), whereas 7 eyes (43.8%) achieved an incomplete VMT separation. Superficial capillary plexus perfusion density was reduced significantly after injection (from 0.427 ± 0.027 to 0.413 ± 0.028; *p* = 0.0146), while no differences were noted in the DCP (*p* = 0.2717) nor in the CC (*p* = 0.6848). Study-eye perfusion density was statistically similar to control eyes in all three plexuses, both at baseline and at follow-up. The FAZ in the SCP area remained unchanged after injection (*p* = 0.168) but was significantly inferior to controls both at baseline and at 1 month (0.198 ± 0.074 vs. 0.196 ± 0.070; *p* = 0.007).

**Conclusions:**

Eyes with VMT have a perfusion density comparable to healthy controls, but a smaller FAZ. After ocriplasmin injection the perfusion density in the SCP is reduced, regardless the anatomical success. Limited by the small sample size and the pilot nature of the study, we found microvascular changes after ocriplasmin injection, which may be due to retinal traction release.

## Background

Age-related posterior vitreous detachment is a physiological process that occurs progressively over time. Incomplete separation of the posterior hyaloid at the macula is termed vitreomacular adhesion (VMA). Continued traction on the macular region without vitreous release can lead to pathologic VMA, producing either vitreomacular traction (VMT), characterized by anatomical distortion of the fovea, or macular hole (MH) formation [1].

Before January 2013, observation and vitrectomy were the only available approaches for patients with VMT [2, 3]. Ocriplasmin (Jetrea; Thrombogenics, Belgium), a truncated form of human serine protease plasmin, is the first approved pharmacological alternative to surgical treatment for vitreomacular traction (VMT), even in association with small or intermediate MH. The enzyme is able to cleave laminin and fibronectin, which are molecules that attach the posterior hyaloid to the retinal surface, with the aim to resolve VMT [4].

Recent optical coherence tomography angiography (OCT-A) studies have shown specific changes that occur in the capillary plexuses of various macular hole subtypes [5–7]. Alterations of the capillary plexuses have also been found in patients affected by idiopathic and secondary epiretinal membrane (ERM), even after surgical removal [8–10]. In addition, recent fluorescein angiography studies show how vitreoretinal traction can alter the microvascular pattern in a reversible fashion, suggesting that there might be a direct mechanical effect of vitreous traction on retinal vascular perfusion [11].

Considering the influence of vitreomacular interface disorders on the retinal capillary plexuses, the rationale of the present study was to use OCT-A to investigate the microvascular changes that occur in patients affected by idiopathic vitreomacular traction (VMT), as well as the possible modifications after intravitreal ocriplasmin injection.

## Methods

In this longitudinal prospective study, we used OCT-A to compare the perfusion density of the superficial capillary plexus (SCP), the deep capillary plexus (DCP), and of the choriocapillaris (CC) before and after intravitreal injection of ocriplasmin for idiopathic VMT. All the data obtained from the investigational group were compared with an age-matched control group.

### Study participants

All subjects were recruited at the Vitreoretinal Surgery Service of the Ophthalmology Department, San Raffaele Scientific Institute between January 2016 and June 2016.

Inclusion criteria were: age ≥ 18 years, axial length < 28 mm (measured with IOL Master, Zeiss), and a diagnosis of idiopathic VMT. The diagnosis was confirmed with spectral domain-optical coherence tomography (SD-OCT).

Exclusion criteria for the study eye were: the presence of ERM, diameter of vitreous attachment > 1500 μm, zonular instability, concurrent ocular disease other than idiopathic VMT involving the posterior segment (e.g. diabetic retinopathy, uveitis, retinal vein occlusion, glaucoma, optic neuropathy, age-related macular degeneration), cataract surgery within 6 months or past complicated cataract surgery, any previous laser or surgical procedure to the posterior segment, previous intravitreal injection of anti-VEGF or steroids. We excluded eyes with media opacities interfering with acceptable quality imaging acquisition. Individuals with systemic vasculopathies (e.g. vasculitides), arterial hypertension, diabetes mellitus, or connective-tissue diseases were also excluded from the study.

### Study protocol

Subjects were consecutively screened during the protocol-recruiting period, and those fulfilling the inclusion/exclusion criteria were enrolled. The study subjects underwent complete ophthalmic examination including: best-corrected visual acuity (BCVA) on Early Treatment Diabetic Retinopathy Study charts, biomicroscopy of the anterior segment, indirect fundus evaluation, SD-OCT and OCT-A scans of the macula. All eyes received intravitreal injection of ocriplasmin (0.125 mg in a 0.1 mL volume) as per the manufacturer’s guidelines. The examination, including OCT-A, was then repeated 1 month after the injection.

A same-size group of healthy subjects was also included in the current analysis as a control. Inclusion criteria were: an unremarkable ophthalmic history, BCVA > 20/20, axial length of 24 ± 0.5 mm, a physiologic optic nerve, normal fundus appearance with SD-OCT within normal limits, no previous surgery other than uncomplicated phacoemulsification. The non-interventional control group underwent two subsets of measurement, one at baseline and the second after 1 month. For size- and age-matching purposes, each control subject was included in a 1:1 proportion with study subjects, with an age equal ±1 to the same study subject.

Owing to the pilot characteristic of this study and to the dearth of any reference data in literature, we considered a convenience patient sample established on a 1-month follow-up. Ocriplasmin is rapidly consumed into the vitreous body, as intravitreal levels reach the lower limits of detection after 7 days [12]. Most of the pharmacodynamic effects are already manifest at 7 days.

The alternative hypothesis H1 postulated a difference in the perfusion density or in the FAZ in any of the three different vascular pleuxes (SCP, DCP, CC). Assuming the low coefficient variation of perfusion density, the study sample size of *N* = 32 allows a test power of 0.85 with an effect size of 1.1, and a significance level of 0.05.

### OCT measurements

SD-OCT and OCT-A scans were obtained with Cirrus HD-OCT (software V.11.0; Carl Zeiss Meditec, Dublin, CA), using both the 3 × 3 and 6 × 6 mm acquisition modules and the follow-up option.

**Fig. 1:**
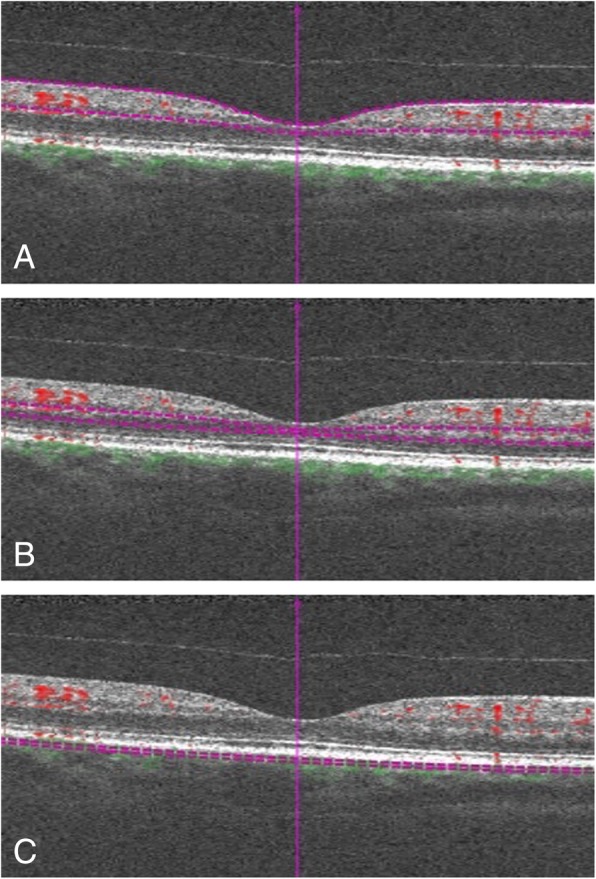
B-scan pictures showing the automatic segmentation of the vascular plexuses. The superficial capillary plexus (**a**) is automatically segmented using as inner boundary the Z_ILM_ (internal limiting membrane layer) and as outer boundary the Z_IPL_ (inner plexiform player). The deep capillary plexus (**b**) inner boundary is Z_IPL_, while the outer is Z_OPL_ (outer plexiform layer). The OPL is estimated as Z_RPE_ (retinal pigment epithelium layer) - 110 μm, and the Z_IPL_ is calculated as: Z_ILM_ + 70%*(T_ILM-OPL_). The choriocapillaris (**c**) inner boundary is Z_RPE_ + 29 μm, and the outer is Z_RPE_ + 49 μm. Violet dashed lines designate the boundaries, while red and green spots respectively represent the perfusion above and below the RPE

One single experienced operator (RC) carried out every examination. In this study, we used only automatically segmented images of the SCP, DCP, and CC to prevent subjective influences in the final analysis. The SCP was automatically segmented using the Z_ILM_ (internal limiting membrane layer) as the inner boundary and the Z_IPL_ (inner plexiform layer) as the outer boundary; the DCP inner boundary was Z_IPL_ while the outer was Z_OPL_ (outer plexiform layer). The OPL was estimated as Z_RPE_ (retinal pigment epithelium layer) - 110 μm, and the Z_IPL_ was calculated as: Z_ILM_ + 70%*(T_ILM-OPL_). The CC inner boundary was Z_RPE_ + 29 μm, and the outer was Z_RPE_ + 49 μm (Fig. 1).

Since any segmentation error would be a significant pitfall for the purpose of this study, the same operator checked each B-scan of every OCT-A acquisition to avoid any rough or subtle segmentation errors, and all inaccurately segmented examinations were excluded. Only good quality and correctly segmented images were included in the analysis.

All OCT-A scans were transferred from the database to ImageJ 1.48 software (National Institutes of Health, Bethesda, Maryland, USA) as a Joint Photographic Experts Group (JPEG) format [13]. The foveal avascular zone (FAZ) area in the SCP was manually delineated using the polygon tool, and was further measured (in mm^2^) using previously published processes [14]. The FAZ was barely recognizable in the DCP, therefore was not included in our evaluation.

**Fig. 2:**
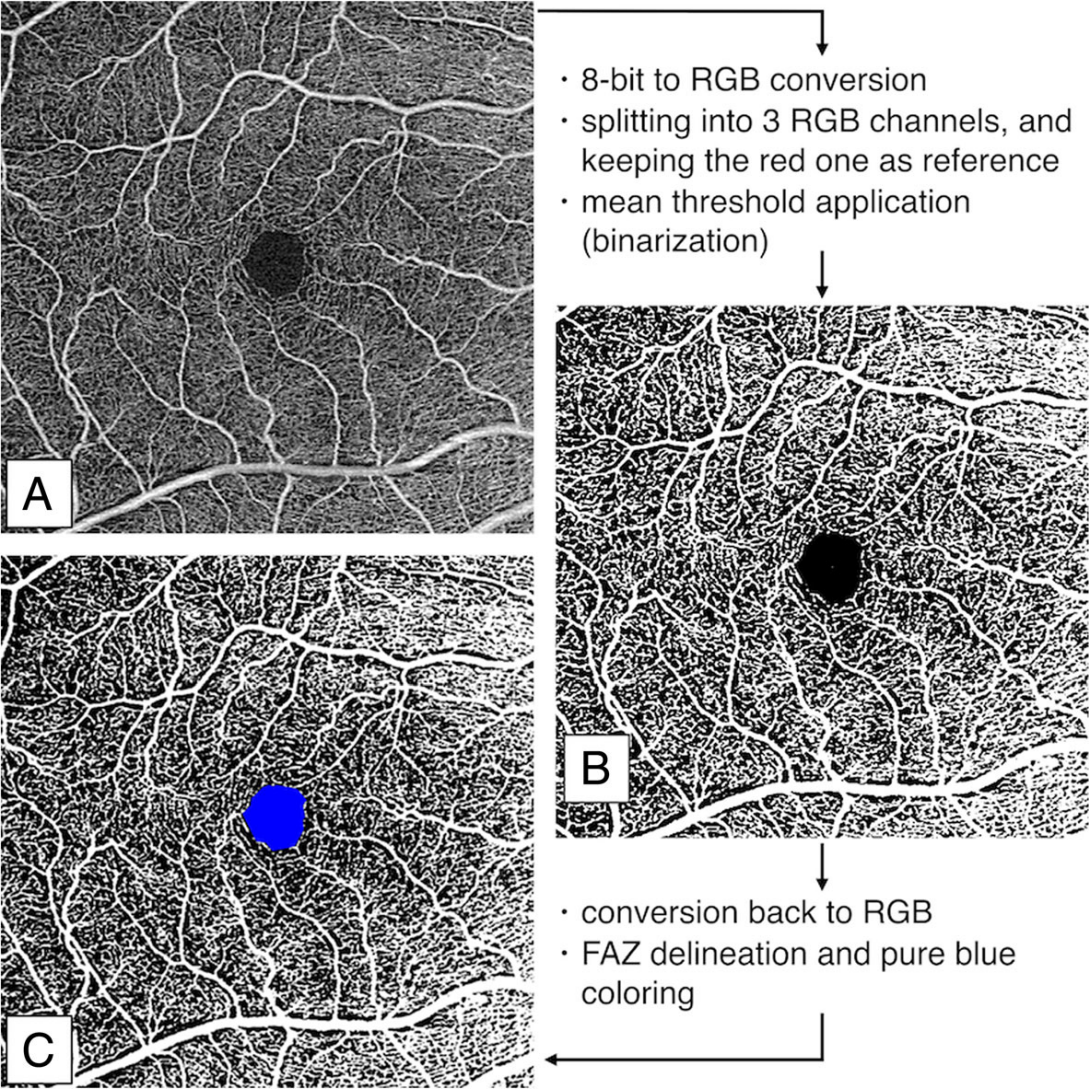
Diagram showing the image analysis procedure of an optical coherence tomography angiography (OCT-A) scan. Binarization example of a 6 × 6 mm OCT-A scan of the superficial capillary plexus of vitreomacular traction (**a**). The procedure consists of (i) 8-bit to red-green-blue (RGB) color-type conversion; (ii) splitting into three channels (RGB) and keeping open the red one as reference; (iii) applying of a mean threshold to convert the image from gray-scale to binary scale (**b**); (iv) re-converting the processed images to RGB; (v) delineating the FAZ area and coloring it with pure blue. After this elaboration (**c**), white pixels represent perfused vessels, black pixels are the background (non-perfused), and blue pixels are automatically excluded from the analysis

To calculate perfusion density, scans were binarized using the threshold strategy [6, 7, 15–18]. Particularly, we fitted a macro that automatically: (i) converts the image from 8-bit to red-green-blue (RGB) type; (ii) separates it into the three channels (red, green, blue), maintaining open the red one as reference; (iii) applies an average fix threshold to convert the image from gray-scale to binary; (iv) converts back the processed scan to RGB; (v) highlights the FAZ area in blue. Following this elaboration, white pixels represent perfused vessels, black pixels are the background (non-perfused), and blue pixels are automatically ignored from the analysis (Fig. 2).

As described by Durbin et al., perfusion density is a variable depicting the total area of perfused vasculature per unit area in a region of interest (ROI), calculated by taking the mean of the binarized slab within a desired region of interest. Consequently, perfusion density was expressed as the ratio between measured vessel pixels and the total scan area after subtracting the FAZ area, thus being a dimensionless quantity [18]. The SCP, DCP, and CC layers of subjects and healthy controls were analyzed using this method.

### Statistical analysis

Statistical analyses were performed using GraphPad Prism version 5.00 for Mac (GraphPad Software, San Diego, California, USA). The Wilcoxon signed rank test was used to compare the outcome measures before and after intervention. The Mann-Whitney test was used to compare investigational results with controls. The Spearman rank correlation test was used to assess the correlation between variables. In all analyses, *p*-values < 0.05 were considered significant.

## Results

We screened a total of 39 eyes with focal VMT. Fourteen eyes were not enrolled in accordance with the exclusion criteria (4 with wet age-related macular degeneration, 3 with diabetic macular edema, 1 with an axial length of 28.8 mm, 6 with concomitant ERM). Nine eyes were excluded after completing the first subset of OCT-A imaging owing to segmentation errors (software inability to correctly recognize the retinal boundaries). We finally analyzed 16 study eyes of 16 subjects (7 males and 9 females, aged 59.2 ± 3.4 years), achieving good quality images.

Out of the 16 fellow-eyes, 3 had idiopathic ERM, 2 had lamellar macular hole, 1 had been operated for full-thickness macular hole 3 years earlier, 2 had received laser photocoagulation for peripheral retinal tear. Eight eyes were clinically normal.

**Fig. 3:**
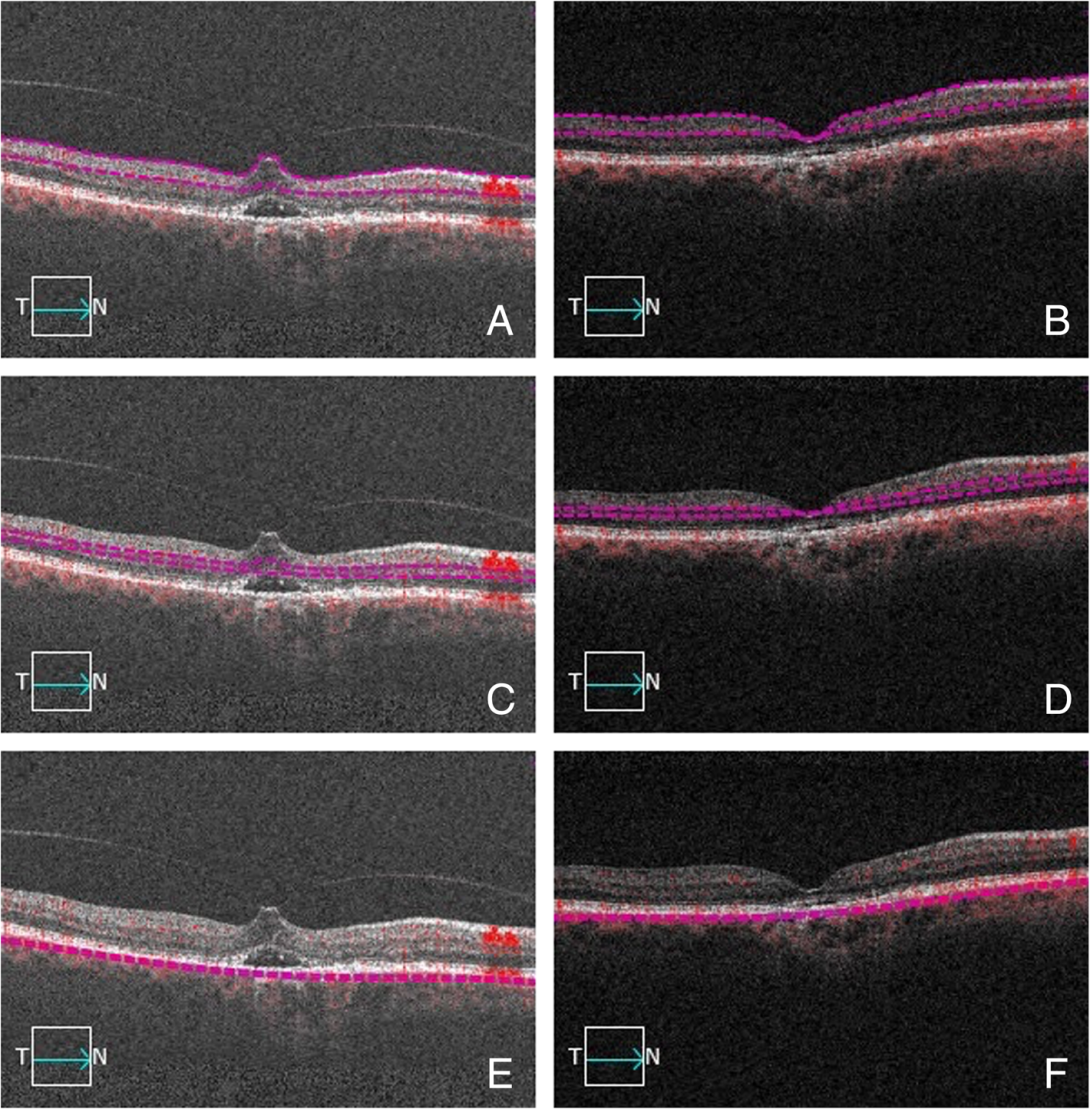
B-scan pictures showing the automatic segmentation of the three vascular plexuses before and after intravitreal ocriplasmin injection in a case achieving complete anatomical resolution. Violet dashed lines designate the correctly-segmented boundaries of the superficial capillary plexus (**a**), deep capillary plexus (**c**) and choriocapillaris (**e**) before ocriplasmin injection. Focal vitreomacular traction with neuroepithelial detachment is evident. Compete vitreomacular traction release is evident after injection (respectively **b**, **d**, **f**)

**Fig. 4:**
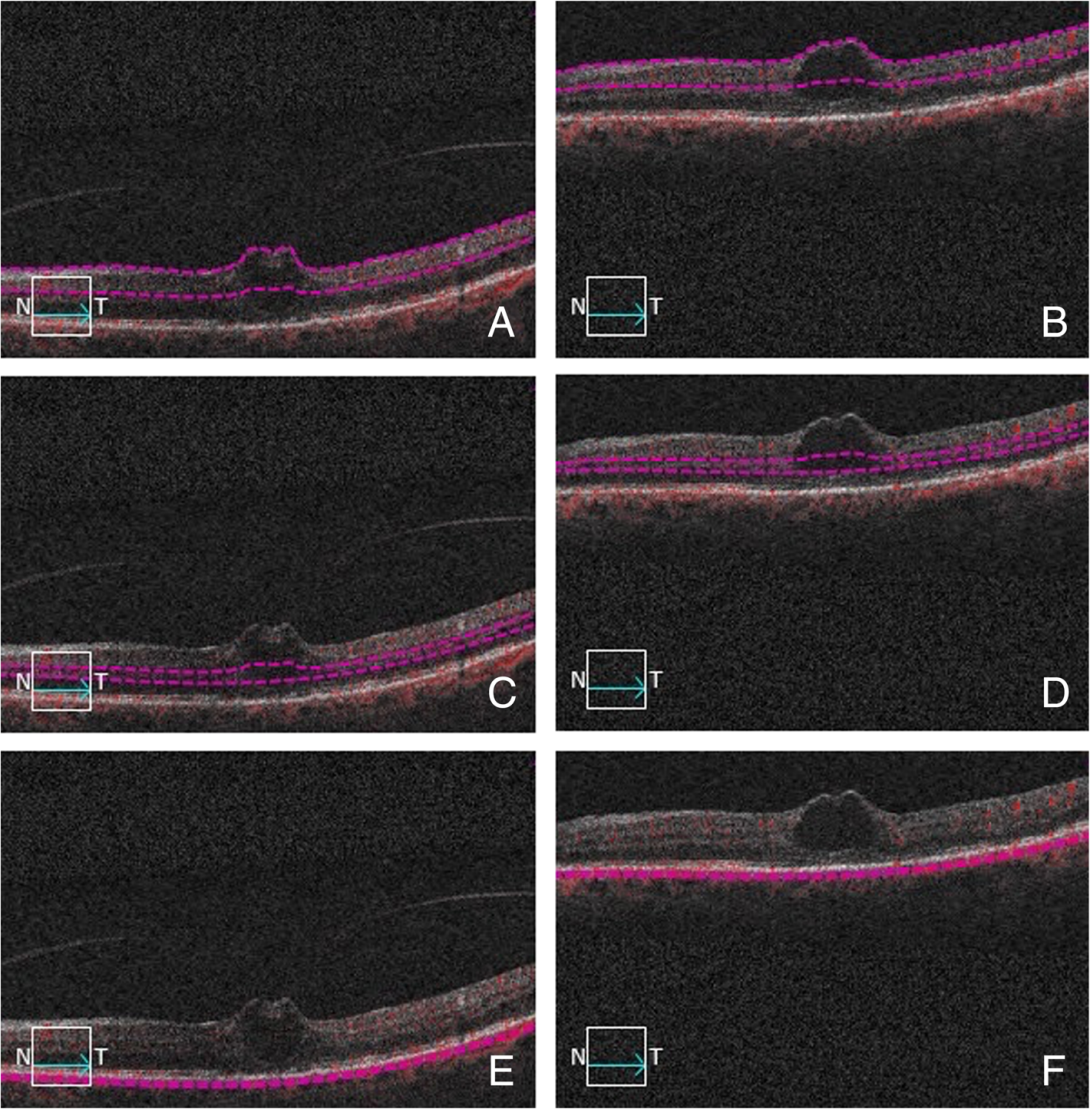
B-scan pictures showing the automatic segmentation of the three vascular plexuses before and after intravitreal ocriplasmin injection in a case revealing vitreomacular traction persistence. Violet dashed lines designate the correctly-segmented boundaries of the superficial capillary plexus (**a**), deep capillary plexus (**c**) and choriocapillaris (**e**) before ocriplasmin injection. Focal vitreomacular traction with intraretinal cysts are evident. Despite persistence of the vitreomacular traction after injection, mild smoothing of macular irregularity is evident (respectively **b**, **d**, **f**)

Sixteen age-matched control eyes of 16 healthy subjects (8 males, 8 females, aged 59.6 ± 1.4) were also included into the analysis. As per protocol, control eyes were also rescanned after one month. The tested variables in the healthy control group (central foveal thickness [CFT], perfusion density and FAZ) all turned out to be stable throughout follow-up (*p* = 0.9999 for CFT, perfusion density and FAZ) (Table 1). Average diagnosis-to-treatment time in the VMT groups was 27.1 ± 6.4 days. Intravitreal injection was performed without any clinically relevant complication in every subject. Complete anatomical resolution of the VMT, judged as complete separation of the posterior hyaloid from the inner retinal surface, was achieved in 9 of the 16 treated eyes (56.2%) (Fig. 3). Seven eyes, despite reduced foveal distortion, showed persistent VMT (Fig. 4). All treated subjects reported a subjective improvement in terms of central scotoma and metamorphopsia.

**Table 1:** Clinical features of the investigational group (vitreomacular traction - VMT) and controls throughout follow-up

	Idiopathic VMT	Control	*p*
Age (years)	59.2 ± 3.4	59.6 ± 1.4	0.998
Sex (f: m)	9:7	8:8	0.999
Axial length (mm)	23.92 ± 0.78	23.85 ± 0.05	0.899
BCVA (logMAR) baseline	0.23 ± 0.04	0.00	< 0.001
BCVA 1 month	0.12 ± 0.03	0.00	< 0.001
CFT baseline	430 ± 98	215 ± 8	< 0.001
CFT 1 month	272 ± 31	216 ± 7	< 0.001

*f* female, *m* male, *BCVA* best corrected visual acuity, *CFT* central foveal thickness, *logMAR* logarithm of the minimal angle of resolution

Clinical outcome measurements are shown in Table 1. Average BCVA significantly increased after injection (*p* = 0.0026), while CFT reduced (*p* = 0.0005). Axial length was similar between the study eye group (23.92 ± 0.78 mm, range 22.93-25.11) and controls (23.85 ± 0.05 mm, range 23.52-24.09; *p* = 0.899).

The slabs of the 3 plexuses (SCP, DCP, CC) did not present significant qualitative or morphological changes after injection in any subject.

**Fig. 5:**
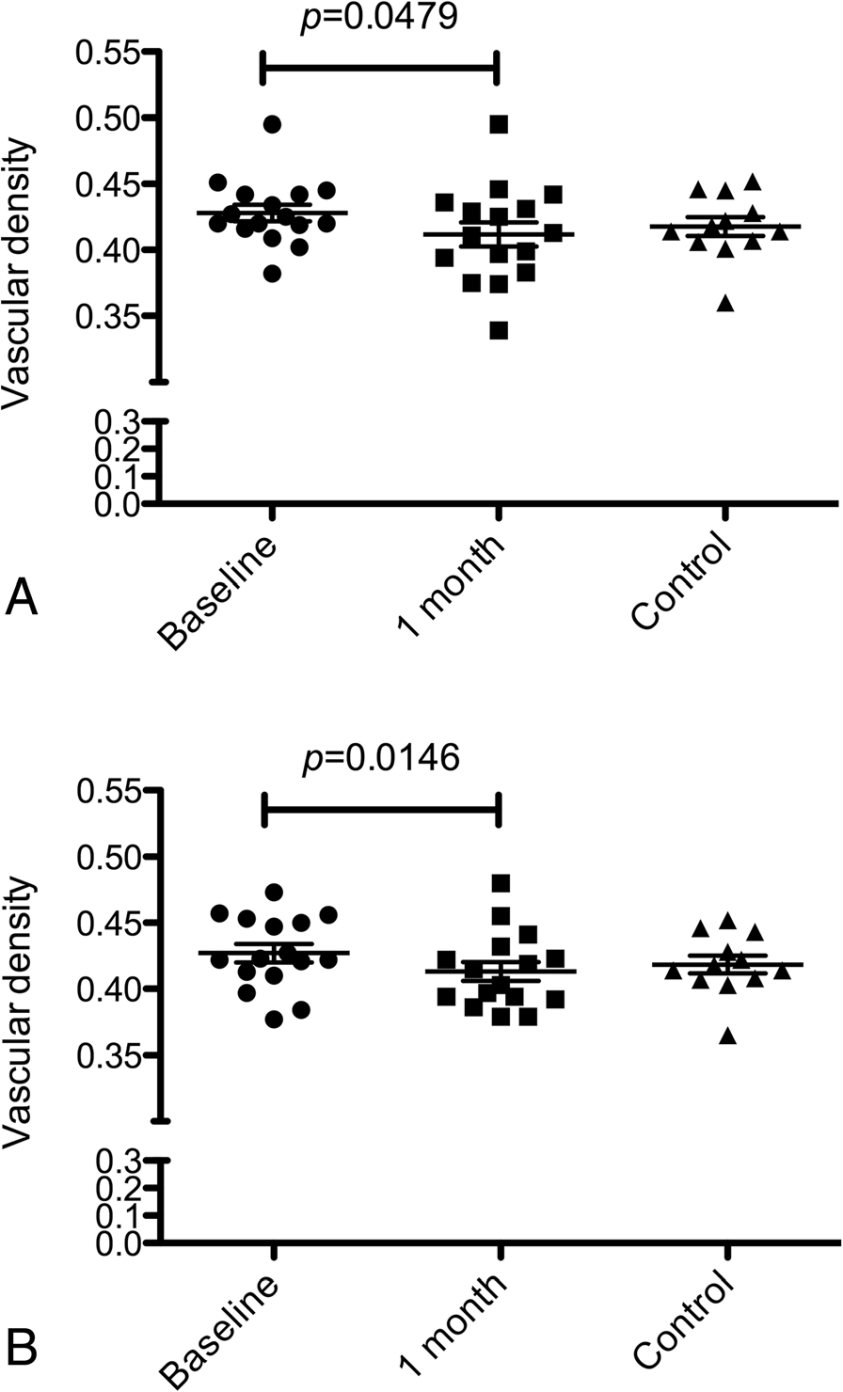
Box-and-whiskers plots of capillary perfusion density in the superficial capillary plexus (SCP). The plots show the perfusion density in the retinal SCP of eyes with idiopathic vitreomacular traction at baseline and at 1 month after ocriplasmin injection, compared with healthy controls. Perfusion density was calculated respectively in the 3 × 3 mm (**a**) and in the 6 × 6 mm (**b**) scans

The perfusion density of the 3 plexuses can be seen in Tables 2 and 3, with 3 × 3 mm scan analysis showing that SCP perfusion density in VMT eyes reduced significantly 1 month after injection (*p* = 0.0479). No post-intervention differences were noted in DCP (*p* = 0.1913) nor in CC (*p* = 0.2441). The 6 × 6 mm analysis revealed similar results: only SCP perfusion density of VMT eyes reduced significantly 1 month after injection (*p* = 0.0146), while no changes were noted in DCP (*p* = 0.2717) nor in CC (*p* = 0.6848) (Fig. 5).

**Table 2:** Perfusion density of the three capillary plexuses (superficial, deep, and choriocapillaris) calculated in the 3 × 3 mm scan area, after subtracting the foveal avascular zone, area in vitreomacular traction (VMT) and control groups

	VMT group			Control		
	Baseline	1 month	*p*	Baseline	1 month	*p*
Vascular density SCP ^a^	0.428 ± 0.025	0.411 ± 0.036	0.0479	0.417 ± 0.024	0.417 ± 0.022	0.9998
Vascular density DCP ^b^	0.391 ± 0.059	0.411 ± 0.029	0.1913	0.428 ± 0.025	0.428 ± 0.026	0.9999
Vascular density CC ^c^	0.483 ± 0.029	0.476 ± 0.029	0.2441	0.501 ± 0.022	0.501 ± 0.025	0.9999

^a^ Superficial capillary plexus

^b^ Deep capillary plexus

^c^ Choriocapillaris plexus

**Table 3:** Perfusion density of the three capillary plexuses (superficial, deep, and choriocapillaris) calculated in the 6 × 6 mm scan area, after subtracting the foveal avascular zone area, in vitreomacular traction (VMT) and control groups

	VMT group			Control		
	Baseline	1 month	*p*	Baseline	1 month	*p*
Vascular density SCP ^a^	0.427 ± 0.027	0.413 ± 0.028	0.0146	0.418 ± 0.023	0.418 ± 0.022	0.9999
Vascular density DCP ^b^	0.434 ± 0.024	0.424 ± 0.025	0.2717	0.429 ± 0.025	0.429 ± 0.027	0.9999
Vascular density CC ^c^	0.472 ± 0.087	0.473 ± 0.026	0.6848	0.500 ± 0.026	0.501 ± 0.025	0.9999

^a^ Superficial capillary plexus

^b^ Deep capillary plexus

^c^ Choriocapillaris plexus

Perfusion density in VMT eyes was statistically similar to the control eyes in each of the three plexuses, both at baseline and at 1 month. Perfusion density in the subgroup of clinically normal fellow-eyes also showed comparable results, being similar to control eyes and to VMT eyes in all three plexuses, both at baseline and at 1 month (Table 4).

**Table 4:** Perfusion density of the three capillary plexuses (superficial, deep, and choriocapillaris) calculated in the 3 × 3 scan area after subtracting the foveal avascular zone area, in the vitreomacular traction (VMT) group according to anatomical success of ocriplasmin injection

	VMT anatomical resolution			VMT persistence		
	Baseline	1 month	*p*	Baseline	1 month	*p*
Vascular density SCP ^a^	0.430 ± 0.021	0.411 ± 0.009	0.0301	0.426 ± 0.025	0.413 ± 0.030	0.0489
Vascular density DCP ^b^	0.393 ± 0.060	0.410 ± 0.028	0.1991	0.391 ± 0.013	0.411 ± 0.036	0.2956
Vascular density CC ^c^	0.483 ± 0.034	0.476 ± 0.037	0.2673	0.483 ± 0.0.26	0.476 ± 0.022	0.3040

^a^ Superficial capillary plexus

^b^ Deep capillary plexus

^c^ Choriocapillaris plexus

Post-injection FAZ area in SCP was unchanged (0.103 ± 0.031 at baseline, 0.115 ± 0.029 at 1 month; *p* = 0.168), but turned out to be significantly less than in healthy controls both at baseline and at 1-month follow-up (0.198 ± 0.074 at baseline, 0.196 ± 0.070 at 1 month; *p* = 0.007).

The FAZ in the SCP did not correlate with 3 × 3 mm-scan perfusion density, neither at baseline (*p* = 0.3790) nor at 1 month (*p* = 0.6239). Similar results were seen in 6 × 6 mm perfusion density scans (*p* = 0.3991 and *p* = 0.5598, respectively).

Perfusion density in both SCP and DCP did not correlate with BCVA at baseline (*p* not significant for all, both 3 × 3 mm and 6 × 6 mm scans). One month after ocriplasmin injection, perfusion density in the 6 × 6 mm slab of SCP showed a moderate degree of correlation with BCVA (r^2^ = 0.4408, *p* = 0.0133). No correlation was found in the 3 × 3 mm slab of SCP, or in any of the DCP slabs (*p* not significant).

The FAZ and the perfusion density were further re-examined, subdividing the study eye population according to axial length ≤ 24.50 mm (10 eyes, 23.43 ± 0.49 mm) and > 24.50 mm (6 eyes, 24.72 ± 0.21 mm). The perfusion density, in each of the three plexuses (SCP, DCP, CC), was similar between the two subgroups, at baseline and 1 month after injection (*p* not significant for all). Analogously, the FAZ measured in the SCP was comparable between the two subgroups at baseline and 1 month after injection (*p* = 0.991).

We further tested whether the rate of VMT anatomical resolution after injection might have influenced the perfusion density change. After treatment, the eyes that achieved anatomical resolution (9 eyes, 56.2%) revealed similar perfusion density compared with eyes where VMT persisted (7 eyes, 43.8%) in all the three vascular layers (SCP, DCP and CC; *p* not significant for all). Retrospectively looking at the same eyes at baseline, similar conclusions were made in the three vascular layers (*p* not significant for all). Perfusion density was confirmed to be significantly reduced in the SCP after injection, in both the 3 × 3 and 6 × 6 scans, either in the VMT resolved group and in the nonresolved (Tables 4 and 5).

**Table 5:** Perfusion density of the three capillary plexuses (superficial, deep, and choriocapillaris) calculated in the 6 × 6 scan area after subtracting the foveal avascular zone area, in the vitreomacular traction (VMT) group according to anatomical success of ocriplasmin injection

	VMT anatomical resolution			VMT persistence		
	Baseline	1 month	*p*	Baseline	1 month	*p*
Vascular density SCP ^a^	0.428 ± 0.027	0.413 ± 0.012	0.0111	0.426 ± 0.031	0.413 ± 0.056	0.0155
Vascular density DCP ^b^	0.434 ± 0.025	0.425 ± 0.018	0.2779	0.434 ± 0.020	0.424 ± 0.029	0.2698
Vascular density CC ^c^	0.472 ± 0.080	0.473 ± 0.031	0.6943	0.472 ± 0.089	0.474 ± 0.023	0.6914

^a^ Superficial capillary plexus

^b^ Deep capillary plexus

^c^ Choriocapillaris plexus

## Discussion

The rationale of our study was to investigate possible microvascular alterations in VMT, and to observe changes after intravitreal ocriplasmin injection. Kashani et al. used fluorescein angiography to show microvascular pattern alterations produced by vitreoretinal traction, and also demonstrated their reversibility, suggesting a possible direct mechanical effect of vitreous traction on retinal perfusion [11].

We used a quantification strategy of OCTA, based on a previously published method [6, 7, 15–17], which allowed us to extract the vascularity coefficient from any analyzed imaged. This pure value represents the total perfused tissue amount extracted from the background tissue.

In our study, we found that eyes affected by idiopathic VMT at baseline have a perfusion density that is quantitatively comparable to healthy controls, yet have a smaller FAZ. After injection, FAZ increased slightly (not significantly), while perfusion density in the SCP was reduced. The other plexuses (DCP and CC) remained stable overall. On note, the vascular changes of the outer retinal layers (DCP), despite not reaching statistical significance, showed a mild trend of reduction in the 6 × 6 mm scans, in agreement with the same findings of the SCP. Perfusion density and FAZ in SCP (where FAZ was identifiable) were statistically independent.

Various hypotheses may be formulated. FAZ area alteration may be due to VMT presence, which mechanically alters the retinal contour by narrowing the foveal margins, together with the microvascular nets. After ocriplasmin injection, mild enlargement of FAZ can be noted.

Although previous studies investigated the FAZ area in separate retinal capillary plexuses (SCP and DCP), more recent papers assess this measurement in a single segment for histologic and technical reasons: (i) a strong body of evidence suggests the retinal plexuses merge at the edge of FAZ, which may thus be considered a singular structure throughout the entire foveal thickness [19]; (ii) assessing FAZ size at different segments may result in increased measurement variability [20].

What is more compelling are the reasons for perfusion density reduction after injection. It has to be considered that, per protocol, ocriplasmin injection is indicated only for focal VMT whose vitreous attachment diameter is < 1500 μm. In the hypothesis of a VMT with a maximal vitreous attachment, its area should roughly measure ≈1.75 mm^2^ (calculated as π × r^2^) [21]. Considering the 36 mm^2^-OCTA scanned area (6 × 6 mm), the maximum VMT surface accounts for less than 5% of the whole scanned macular area. The OCT-A scan therefore includes a retinal surface significantly wider than the sole VMT area, and this larger region is less likely to be influenced by segmentation errors caused by VMT-induced retinal distortion. Moreover, our measurement protocol assumed the calculation of perfusion density in the scanned are after subtracting the FAZ, being so a true peri-FAZ density. Therefore, it might be speculated that the hyaloid, despite eliciting a visible effect on the fovea, draws mechanical stress onto a wider macular region, which is reflected in the retinal microvasculature as vascular engorgement. This change might produce the observed SCP perfusion density increase. Similar conclusions were also reported by Pierro [6, 7]. The post-ocriplasmin VMT release (complete or incomplete) might justify the observed tissue relaxation, together with microvascular density reduction. This hypothesis might consolidate the findings made by Kashani [11], and Mastropasqua [8].

Recently, certain remarks regarding the possible interaction/toxic effect of ocriplasmin on the retinal architecture have been waived. Being a small (27.2 kDa) recombinant protein, ocriplasmin can penetrate all retinal layers, and has been shown in animal models to degrade fibronectin and laminin at the outer retinal layers, specifically on the extracellular matrix and/or basement membranes, without photoreceptor digestion [22]. However, some laminin isoforms have been identified in the endothelial and perivascular basement membrane [23]. Indeed, various studies report OCT alterations after ocriplasmin therapy in a substantial number of eyes (17–56% of cases), and that these transient changes are particularly prominent in the ellipsoid zone (EZ) [24, 25]. In addition, diffuse retinal vascular constriction/attenuation has been reported after ocriplasmin injection in both human and animal models [26, 27]. It cannot be excluded that the small change in the perfusion density we observed with OCT-A might be a manifestation of the adverse acute enzymatic effect of ocriplasmin on the retina and its vasculature.

Since we are unable to ascertain whether these changes are due to traction release or to the direct effect of ocriplasmin on the retinal architecture, our considerations remain speculative. Given the absence of long prospective follow-up cohort studies (especially regarding OCT-A prognostic value on treated and untreated eyes), this slight post-injection microvascular change cannot be considered clinically relevant.

Furthermore, we cannot exclude that similar results may occur in eyes experiencing a spontaneous VMT resolution or in eyes treated with sham injections. Such cases might bring evidence that the microvascular changes were only triggered by the VMT and rule out any influence of a drug-related effect.

The functional variable (BCVA) was also reported, although the injection success was not meant to be judged according to functional improvement. Indeed, there are controversial cases where BCVA improves despite VMT persistence, or cases where BCVA does not increase during VMT resolution. Although we found an average BCVA improvement, post-injection microvascular changes were independent from the functional change. We only found a moderate correlation between BCVA improvement and perfusion density reduction in the 6 × 6 mm slab of SCP after treatment.

We acknowledge that our study has several limitations, mainly due to the small sample size, the short follow-up, and the possible flaws of OCT-A software analysis. Despite manual checking for segmentation errors, we cannot exclude that automated segmentation might have produced significant inaccuracy, such as in case of cysts or foveal detachment. However, both the exclusion of the FAZ from perfusion calculation, and the relatively low impact of the VMT area onto the wider OCT-A scanned area together account for a significant consistency of the measured changed. In addition, although we adopted different enrollment criteria regarding axial length (< 28 mm for study eyes, 24 ± 1 mm for controls), this variable was comparable between the two groups. The 28 mm value was only considered to fit ocriplasmin manufacturer indications. However, a sub-analysis comparing eyes with axial length ≤ vs > 24.50 mm, ruled out any possible influence of axial length on FAZ and perfusion density within our study eyes.

Another sub-analysis, grouping eyes according to the rate of anatomical success after injection, despite reaching the same conclusion of the whole eye population, showed a mild trend of increased changes in the group that achieved a complete anatomical resolution (Tables 4 and 5). These date are in agreement with the tractional hypothesis responsible for the perfusion density change.

Strengths of our study are the accurate eye selection, as we included only idiopathic VMT and excluded patients with vascular comorbidities (whose results could have jeopardized our results). The control group was rescanned throughout the follow-up to exclude other biasing external factors (repeatability biases). Of note, data regarding the variability of perfusion density and FAZ within a brief time-interval have been reported to have high levels of repeatability and reproducibility, both for healthy subjects and eyes affected by various retinal diseases [28–30]. For further empowered potential follow-up studies, we calculated to include 64 subjects (that might be feasible with size- and center-doubling) to achieve a 30% reduction of effect size with a power of 0.8.

The healthy control group may also be considered a limitation, as it might be better to include the fellow-eye as reference (reducing biological variables among individuals). We intentionally decided not to include the fellow-eye in this study for two reasons: firstly, the prevalence of a concomitant vitreoretinal interface disorder in the fellow-eye is relatively high (as seen in our data, 50% of fellow-eyes); secondly, it has been shown that even clinically healthy and OCT-silent fellow-eyes can show early involvement of the retinal capillary plexuses [6]. Furthermore, it might be questioned whether other diseases (e.g. the spectrum of vitreoretinal interface disorders) might be more suitable as controls. Since no data regarding the OCT-A characteristics of idiopathic VMT are available, nor there are data on the possible changes after ocriplasmin injection, we preferred to compare our results with a healthy-eye population. This choice was made to avoid possible flaws resulting from a heterogeneous population of fellow-eyes, and to obtain a reference for future comparative studies (e.g. with other diseases).

Despite these limitations, and the fact that our conclusions are merely speculative, we believe our work gives new insights into the effects and safety of ocriplasmin. Further aspects, particularly concerning OCT-A prognostic value, should certainly be considered for correct pathophysiological interpretation, and for a comprehensive analysis of this clinical aspect.

## Conclusion

Our research was intended to investigate, using OCT-A, the microvascular features of eyes with idiopathic VMT, and to evaluate the changes occurring after ocriplasmin injection. We found these eyes have a perfusion density comparable to healthy controls, but a smaller FAZ. After ocriplasmin injection the perfusion density in the SCP is reduced, regardless the anatomical success. Microvascular changes are hence evident after ocriplasmin injection, which we hypothesize may be due to the partial or incomplete retinal traction release.

The study emphasizes the clinical relevance of the vitreoretinal interface on the retinal vascularization, thereby providing adjunctive data about the effects and safety of ocriplasmin.

## Abbreviations

BCVA: Best-corrected visual acuity
CC: Choriocapillaris
CFT: Central foveal thickness
DCP: Deep capillary plexus
ERM: Epiretinal membranes
FAZ: Foveal avascular zone
MH: Macular hole
OCT-A: Optical coherence tomography angiography
SCP: Superficial capillary plexus
SD-OCT: Spectral domain-optical coherence tomography
VEGF: Vascular endothelial growth factor
VMA: Vitreomacular adhesion
VMT: Vitreomacular traction

## References

[1] Duker JS, Kaiser PK, Binder S, de Smet MD, Gaudric A, Reichel E (2013). The international Vitreomacular traction study group classification of vitreomacular adhesion, traction, and macular hole. Ophthalmology..

[2] Odrobina D, Michalewska Z, Michalewski J, Dzięgielewski K, Nawrocki J (2011). Long-term evaluation of vitreomacular traction disorder in spectral-domain optical coherence tomography. Retina..

[3] Jackson TL, Nicod E, Angelis A, Grimaccia F, Prevost AT, Simpson ARH (2013). Pars plana vitrectomy for vitreomacular traction syndrome: a systematic review and metaanalysis of safety and efficacy. Retina..

[4] Stalmans P, Benz MS, Gandorfer A, Kampik A, Girach A, Pakola S (2012). Enzymatic vitreolysis with ocriplasmin for vitreomacular traction and macular holes. N Engl J Med.

[5] Pierro L, Iuliano L, Bandello F (2016). OCT angiography features of a case of bilateral full-thickness macular hole at different stages. Ophthalmic Surg Lasers Imaging Retina..

[6] Pierro L, Rabiolo A, Iuliano L, Gagliardi M, Panico D, Bandello F (2017). Vascular density of retinal capillary plexuses in different subtypes of macular hole. Ophthalmic Surg Lasers Imaging Retina.

[7] Pierro L, Iuliano L, Gagliardi M, Arrigo A, Bandello F (2019). Higher vascular density of the superficial retinal capillary plexus in degenerative lamellar macular holes. Ophthalmic Surg Lasers Imaging Retina..

[8] Mastropasqua L, Borrelli E, Carpineto P, Toto L, Di Antonio L, Mattei PA, et al. Microvascular changes after vitrectomy with internal limiting membrane peeling: an optical coherence tomography angiography study. Int Ophthalmol. 2017. 10.1007/s10792-017-0608-1 28631180

[9] Romano MR, Cennamo G, Schiemer S, Rossi C, Sparnelli F, Cennamo G (2017). Deep and superficial OCT angiography changes after macular peeling: idiopathic vs diabetic epiretinal membranes. Graefes Arch Clin Exp Ophthalmol.

[10] Nelis P, Alten F, Clemens CR, Heiduschka P, Eter N (2017). Quantification of changes in foveal capillary architecture caused by idiopathic epiretinal membrane using OCT angiography. Graefes Arch Clin Exp Ophthalmol.

[11] Kashani AH, Zhang Y, Capone A, Drenser KA, Puliafito C, Moshfeghi AA (2016). Impaired retinal perfusion resulting from vitreoretinal traction: a mechanism of retinal vascular insufficiency. Ophthalmic Surg Lasers Imaging Retina..

[12] Syed YY, Dhillon S (2013). Ocriplasmin: a review of its use in patients with symptomatic vitreomacular adhesion. Drugs..

[13] Schneider CA, Rasband WS, Eliceiri KW (2012). NIH image to ImageJ: 25 years of image analysis. Nat Methods.

[14] Samara WA, Say EAT, Khoo CTL, Higgins TP, Magrath G, Ferenczy S (2015). Correlation of foveal avascular zone size with foveal morphology in normal eyes using optical coherence tomography angiography. Retina..

[15] Nemiroff J, Kuehlewein L, Rahimy E, Tsui I, Doshi R, Gaudric A (2016). Assessing deep retinal capillary ischemia in paracentral acute middle maculopathy by optical coherence tomography angiography. Am J Ophthalmol.

[16] Chidambara L, Gadde SGK, Yadav NK, Jayadev C, Bhanushali D, Appaji AM (2016). Characteristics and quantification of vascular changes in macular telangiectasia type 2 on optical coherence tomography angiography. Br J Ophthalmol.

[17] Shahlaee A, Samara WA, Hsu J, Say EAT, Khan MA, Sridhar J (2016). In vivo assessment of macular vascular density in healthy human eyes using optical coherence tomography angiography. Am J Ophthalmol.

[18] Durbin MK, An L, Shemonski ND, Soares M, Santos T, Lopes M (2017). Quantification of retinal microvascular density in optical coherence tomographic angiography images in diabetic retinopathy. JAMA Ophthalmol..

[19] Gariano RF, Iruela-Arispe ML, Hendrickson AE (1994). Vascular development in primate retina: comparison of laminar plexus formation in monkey and human. Invest Ophthalmol Vis Sci.

[20] Coscas F, Sellam A, Glacet-Bernard A, Jung C, Goudot M, Miere A (2016). Normative data for vascular density in superficial and deep capillary plexuses of healthy adults assessed by optical coherence tomography angiography. Invest Ophthalmol Vis Sci.

[21] Codenotti M, Iuliano L, Fogliato G, Querques G, Bandello F (2014). A novel spectral-domain optical coherence tomography model to estimate changes in vitreomacular traction syndrome. Graefes Arch Clin Exp Ophthalmol.

[22] Chen W, Mo W, Sun K, Huang X, Zhang Y, Song H (2009). Microplasmin degrades fibronectin and laminin at vitreoretinal interface and outer retina during enzymatic vitrectomy. Curr Eye Res.

[23] Yousif LF, Di Russo J, Sorokin L (2013). Laminin isoforms in endothelial and perivascular basement membranes. Cell Adhes Migr.

[24] Itoh Y, Ehlers JP (2016). Ellipsoid zone mapping and outer retinal characterization after intravitreal ocriplasmin. Retina..

[25] Quezada-Ruiz C, Pieramici DJ, Nasir M, Rabena M, Steinle N, Castellarin AA (2015). Outer retina reflectivity changes on sd-oct after intravitreal ocriplasmin for vitreomacular traction and macular hole. Retina..

[26] Fahim AT, Khan NW, Johnson MW (2014). Acute panretinal structural and functional abnormalities after intravitreous ocriplasmin injection. JAMA Ophthalmol..

[27] Johnson MW, Fahim AT, Rao RC (2015). Acute ocriplasmin retinopathy. Retina..

[28] Lei J, Durbin MK, Shi Y, Uji A, Balasubramanian S, Baghdasaryan E (2017). Repeatability and reproducibility of superficial macular retinal vessel density measurements using optical coherence tomography angiography En face images. JAMA Ophthalmol.

[29] Mihailovic N, Brand C, Lahme L, Schubert F, Bormann E, Eter N (2018). Repeatability, reproducibility and agreement of foveal avascular zone measurements using three different optical coherence tomography angiography devices. PLoS One.

[30] Czakó C, Sándor G, Ecsedy M, Récsán Z, Horváth H, Szepessy Z (2018). Intrasession and between-visit variability of retinal vessel density values measured with OCT angiography in diabetic patients. Sci Rep.

